# Characterization of novel *Acidobacteria* exopolysaccharides with potential industrial and ecological applications

**DOI:** 10.1038/srep41193

**Published:** 2017-01-24

**Authors:** Anna M. Kielak, Tereza C. L. Castellane, Joao C. Campanharo, Luiz A. Colnago, Ohana Y. A. Costa, Maria L. Corradi da Silva, Johannes A. van Veen, Eliana G. M. Lemos, Eiko E. Kuramae

**Affiliations:** 1 Netherlands Institute of Ecology (NIOO-KNAW), Department of Microbial Ecology, P.O. Box 50, 6700 AB, Wageningen, the Netherlands; 2 São Paulo State University (Unesp), School of Agricultural and Veterinarian Sciences, Rod. Prof. Paulo Donato Castellane km 5, CEP 14884-900, Jaboticabal, Brazil; 3 Embrapa Instrumentação, Rua XV de Novembro, 1452, CEP 13560-970, São Carlos, Brazil; 4 Faculdade de Ciências e Tecnologia (Unesp), Departmento de Química e Bioquímica, Rua Roberto Símonsen, 305, CEP 19060-900, Presidente Prudente, Brazil

## Abstract

*Acidobacteria* have been described as one of the most abundant and ubiquitous bacterial phyla in soil. However, factors contributing to this ecological success are not well elucidated mainly due to difficulties in bacterial isolation. *Acidobacteria* may be able to survive for long periods in soil due to protection provided by secreted extracellular polymeric substances that include exopolysaccharides (EPSs). Here we present the first study to characterize EPSs derived from two strains of *Acidobacteria* from subdivision 1 belonging to *Granulicella* sp. EPS are unique heteropolysaccharides containing mannose, glucose, galactose and xylose as major components, and are modified with carboxyl and methoxyl functional groups that we characterized by Fourier transform infrared (FTIR) spectroscopy. Both EPS compounds we identified can efficiently emulsify various oils (sunflower seed, diesel, and liquid paraffin) and hydrocarbons (toluene and hexane). Moreover, the emulsions are more thermostable over time than those of commercialized xanthan. Acidobacterial EPS can now be explored as a source of biopolymers that may be attractive and valuable for industrial applications due to their natural origin, sustainability, biodegradability and low toxicity.

*Acidobacteria* is a very abundant and ubiquitous bacterial phylum in natural ecosystems^1^,^2^,^3^,^4^. It has been suggested that exopolysaccharide (EPS)-producing bacteria may be able to survive for long periods in soil due to protective properties of EPS. The dominance of *Acidobacteria* in acidic environments and chemically polluted sites (*e.g.* where heavy metal^5^,^6^,^7^, petroleum compounds^8^, linear alkylbenzene sulfonate^9^ and p-nitrophenol^10^ are major contaminants) is related to the ability of these bacteria to produce large amounts of EPS.

The ability to synthesize EPS has been previously reported for some members of the *Acidobacteria* phylum. These reports include genome mining studies^11^, description of cultured *Acidobacteria* species^12^,^13^ and their interactions with plants^14^. However, EPS was never isolated and characterized in these studies. It was suggested that EPS may provide protection against environmental stress and enable bacterial survival under unfavourable soil conditions, since *Acidobacteria* are abundant^15^. The potential importance of EPS in soil is great - it may be involved in the formation of soil matrix, serve to sequester water and nutrition, and be involved in bacterial cell-surface adherence and soil aggregate formation^12^. These hypotheses have not yet been confirmed by functional studies and such functions may overlap with general biological and ecological functions that are protective in nature for *Acidobacteria*.

The ability to synthesize EPS and secrete it into the environment as soluble (slime) or insoluble (capsular) polymers is quite common among bacteria^16^. However, EPSs produced by various bacteria vary in molecular weight, composition and physicochemical properties that include gelling, emulsifying, stabilizing, thickening, suspending, coagulating and texture-enhancing. Though, only few of these bacterial products (*e.g.* alginate, dextran, gellan gum, xanthan) have been successfully commercialized.

Biopolymers including novel microbial-derived EPSs are very attractive for the industrial sector due to their natural origins, sustainability, biodegradability and general low toxicity^17^. Industrial applications of microbial EPSs have been extensively reviewed^17^,^18^,^19^,^20^,^21^,^22^,^23^. The broad spectrum of applications for EPSs range from human health, to food and fodder production, to chemical industry and environmental technologies (*e.g.* bioremediation and phytoremediation).

In the food industry, EPSs are used as thickening, gelling and suspending agents. For example, xanthan (from *Xanthomonas campestris*) is used as food additive^16^ (European Union Food Safety Authority, food additive E415). The EPSs also have the properties as bioemulsifiers that are used in the cosmetic and chemical (*e.g.* pesticide) industries and for bioremediation of soils and water by enhancing oil and heavy metal recovery^24^.

In light of the major absence of published reports on acidobacterial EPS, the aim of the present study was to gain insights into the physicochemical nature of EPS polymers produced by two genetically closely related *Acidobacteria* strains belonging to *Granulicella* sp., which genomes are not sequenced. Moreover, this research focused on the capacity of these EPSs on emulsion formation and stability, which are important properties for industrial and ecological applications.

## Results and discussion

### EPS production by acidobacterial isolates

Different bacterial species produce different extracellular matrices in different bacterial growth phases. For example, cellulose, gellan and alginate are exclusively produced by *Azotobacter vinelandii* during the exponential growth phase, curdlan by *Alcaligenes faecalis* during the deceleration growth phase, xanthan by *Xanthomonas campestris* during the exponential and stationary phases^25^,^26^, and EPSs are produced by *Alteromonas macleodii* from the end of the exponential phase through the stationary phase^27^. In the present study, the yield of EPS produced by *Granulicella* spp. WH15 and 5B5 strains increases in the late growth stage (data not shown). The EPS yield was much greater on solid medium than in liquid culture. Therefore, the fifth day of bacterial growth on solid medium was selected for EPS extraction from both bacterial strains. Strain WH15 showed greater production of EPS than strain 5B5 under experimental conditions (90 mm-diameter Petri dish): 36.95 mg (dry weight) and 9.56 mg per plate, respectively. Different yields of EPS from closely related isolates belonging to the same genus have also been reported for various bacterial taxonomic groups (*e.g. Rhizobium*^28^, *Halomonas*^29^ and *Xanthomonas*^30^).

Both acidobacterial exopolysaccharides were soluble in water and insoluble and stable in all tested organic solvents (chloroform, methanol and toluene).

### EPS Component Analysis

Bacterial extracellular matrix chiefly consists of polysaccharides. Other compounds including proteins, nucleic acids, lipids and humic acids comprise up to 40% of EPS content^31^. However, in this study our main interest was on carbohydrates, therefore we focused on the major carbohydrate fraction of acidobacterial EPS after removing insoluble particles by downstream extraction processes.

A robust and simple extraction method^32^ allowed us to isolate and purify EPS from acidobacterial cultures. This resulted in high quality extracts with very low amounts of proteins. We refer to EPS produced by *Granulicella* sp. strains WH15 and 5B5 as EPSWH15 and EPS5B5, respectively. No protein was detected in EPSWH15, whereas the protein content in EPS5B5 was not significant (<0.01% (w/w) protein relative to total EPS extract). The two EPS preparations differed slightly in total carbohydrate content: 43.5% ± 3.9% (s.d.) for EPSWH15 and 41.8% ± 2.0% for EPS5B5. However, this difference is not statistically significant (*p* > 0.05). The fractional carbohydrate composition of our EPS extracts was considerably greater than that previously reported for a *Pseudomonas* sp. (33.81%) content^33^. It has been suggested that the differences in determined carbohydrate mass might be attributed to residual water of hydration as EPS extract is composed of 99% water^34^,^35^.

### Structural analysis of EPS - molecular weight, compositional homogeneity and monomeric composition

The size homogeneities and molecular weights of acidobacterial EPSWH15 and EPS5B5 were determined using high-performance size-exclusion chromatography (HPSEC) and (Fig. S1). The molecular weights were estimated to be greater than 1.4 × 10^6^ Da according to a standard calibration curve obtained for various molecular weight dextran standards. The degree of polydispersity (currently defined by the IUPAC as dispersity, D_M_ = M_w_/M_n_, where M_w_ is the weight-average molar mass and M_n_ is the number-average molar mass) was 1.4 and 1.3 for EPSWH15 and EPS5B5, respectively, suggesting relatively uniform dispersity (homogeneous size distribution).

We determined the monomeric saccharide composition for the exopolysaccharides after acid hydrolysis (Table 1). Both EPSs were identified as heteropolysaccharides containing mannose (Man), glucose (Glc), galactose (Gal) and xylose (Xyl) as major components in relative molar proportions of 1.0/1.3/2.5/4.1 (Man/Glc/Gal/Xyl) for EPSWH15 and 1.0/0.1/0.6/0.3 for EPS5B5. Additionally, rhamnose (Rha), glucuronic (GlcA) and galacturonic (GalA) acids were identified as minor components of EPSWH15, whereas EPS5B5 contained slightly more Rha than Glc, GlcA was a minor component and GalA was absent to the limit of measurement sensitivity (Table 1). However, the quantities of GlcA and GalA might be underestimated due to the hydrolyses conditions used in this study, which are efficient for hexoses, pentoses and deoxy sugars but not ideal for uronic acids. The monosaccharide compositions we determined for EPSWH15 and EPS5B5 are in basic agreement with previously published observations^36^ since mannose (Man), glucose (Glc), galactose (Gal) and xylose (Xyl) are among the most common monosaccharides found in EPS^37^,^38^.

**Figure 1:**
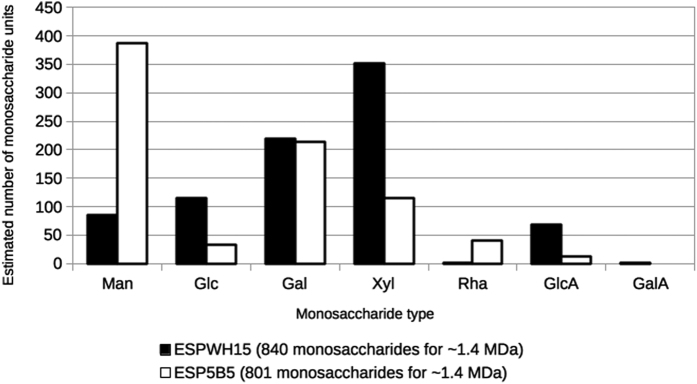
Monosaccharide composition of EPSWH15 (black filled bars) and EPS5B5 (white filled bars) modelled using experimentally measured EPS monomer masses (Mw) and relative monosaccharide abundances determined in the present study. Monosaccharide types: mannose, Man; glucose, Glc; galactose, Gal; xylose, Xyl; rhamnose, Rha; glucuronic acid, GlcA; galacturonic acid, GlaA. For each ~1.4 MDa sized EPS, the approximate number of monosaccharides for each monosaccharide type was calculated according to the equation: 1,400,000 (Da) ≤ n[Σ(Mi)(ai)], where n is an integer multiplicative factor (the independent variable), and Mi and ai are the molecular mass and experimentally determined relative abundance, respectively, for each monosaccharide type i.

Having experimentally determined the monomeric saccharide compositions and the mean molecular masses of EPSWH15 and EPS5B5, and having additionally determined that their measured dispersities suggest relatively homogeneous size distributions, we roughly model the number of constituent monomer units of each monosaccharide type for each monomeric EPS molecule. Accordingly, for ~1.4 kDa sized EPS monomers, EPSWH15 is expected to be comprised of 86 Man, 114 Glc, 219 Gal, 351 Xyl, 1 Rha, 68 GlcA and 1 GalA monosaccharides (Fig. 1). EPS5B5 is expected to be comprised of 387 Man, 33 Glc, 214 Gal, 115 Xyl, 40 Rha, 12 GlcA and no GalA monosaccharides. Thus, while both EPSs contain roughly equal amounts of Gal, EPSWH15 is enriched ~3-fold for Xyl relative to EPS5B5, while EPS5B5 is enriched ~4-fold for Man relative to EPSWH15. Of the monosaccharide types occurring in relatively lower abundances for both EPSs, EPSWH15 is enriched ~3-fold for Gal and ~5.7-fold for GlcA relative to EPS5B5, whereas EPS5B5 is enriched ~40-fold for Rha relative to EPSWH15. Additionally, only EPSWH15 was found to contain detectable GalA (~1 monosaccharide per 1.4 kDa monomer). Interestingly, Xyl is common in eukaryotic polysaccharides, but less commonly occurring in bacteria^39^. Xyl was previously identified, for example, in EPSs of *Zoogloea* sp.^40^, *Myxococcus xanthus, Pseudomonas solanacearum*^41^, *Alteromonas hispanica*^39^ and *Acidithiobacillus ferrooxidans*^42^.

### FTIR spectral analysis of EPS functional groups

Chemical composition, type of glycosides linkage and branching of polysaccharides are factors that affect the overall structure of polysaccharides. In addition to structure, these factors determine the physicochemical properties of polysaccharides, which together with 3-dimensional structure impact physiological functions^16^. Infrared (IR) spectroscopy by Attenuated Total Reflectance (ATR) allows the identification of molecular bonds by measuring the absorption of light at a given wavelength (λ) or wave number (1/λ). We collected ATR-FTIR spectra of EPSWH15 and EPS5B5 (Fig. S2). Both EPSs showed strong absorption bands in the range of 4000 to 400 cm^−1^. The spectra between 3600 to 3200 cm^−1^ of EPSWH15 and EPS5B5 indicate to stretching vibration of O-H^43^. The absorption peak from 3000 cm^−1^ to 2900 cm^−1^ is attributed to the C-H stretching and bending vibrations^44^. The peaks in the range between 1640 to 1550 cm^−1^ and 1460 to 1400 cm^−1^ are absorption bands of carboxylate groups, including a set of strong C-O stretching bands at 1456 and 1450 cm^−1^. In this case, the peak can be also attributed to stretching of the carboxylate functional group (COO-), consistent with the EPSs being acidic polysaccharides. In the ATR-FTIR spectra of EPSWH15 and EPS5B5, there were also signals characterizing unionized carboxylic groups and bands for the respective O–H stretching at 1641 and 1658 cm^−1^, respectively. In the ATR-FTIR spectra of EPS5B5, there were no peaks around 1740 cm^−1^, which are attributed to C = O stretching of carbonyls in esters. In contrast, for EPSWH15, there is a very low band intensity at 1766 cm^−1^ corresponding to C = O stretching of carbonyls in esters (Fig. S2a).

Other signals at 1100 and 1050 cm^−1^ indicate glucopyranosyl residues. A complex sequence of peaks are observed in the region from 1200 to 800 cm^−1^, due in part to C-O-C, C-O, ring-stretching vibrations of polysaccharides^45^. This spectral domain is considered the fingerprint region of EPS, and can be used to assign α and β glycosidic linkages. Both EPSs showed bands at ~820 cm^−1^ and ~890 cm^−1^, characteristic of α and β glycosidic linkages, respectively.

### Nuclear magnetic resonance (NMR) spectroscopy analysis of EPS

We conducted ^1^H NMR and ^13^C NMR studies in order to confirm the presence of functional groups and monosaccharide constituents for both EPSs (Fig. S3). Hydrolysates of EPSs produced by both *Granulicella* strains were first analysed by ^1^H NMR spectroscopy. The ^1^H NMR spectrum of the EPS showed the presence of pyruvate groups as revealed by resonances at 2.22 ppm. Presence of ketal-linked pyruvate and glucuronic acid or galacturonic acid has been reported to be responsible for the polyanionic nature of the exopolysaccharides^17^. Additionally, this spectrum showed some proton resonances belonging to CH_2_ or CH_3_ groups. In Figure S3a, all of the signals in the ^1^H NMR spectra could be assigned to the various constituent monosaccharides, including glucose, rhamnose, mannose, galactose, and xylose, confirming that the EPSs are acidic heteropolysaccharides. ^1^H NMR and ^13^C NMR spectra taken together led us to conclude the absence of acetate groups (Fig. S3a, S3b).

The ^13^C NMR spectra (Fig. S3b) from 5B5 (Fig. S3b1) and WH15 (Fig. S3b2) showed four specific regions corresponding to carbohydrates. The signals at high field around 20 ppm indicate the presence of a methyl group from rhamnose, a deoxy sugar, and from pyruvic acid. Two bands of more intense signals in the region of anomeric carbons at 98 and 104 ppm indicate, respectively, monomer units involved in α and β glycosidic linkages. These data confirm our conclusions from the ATR-FTIR analysis. Signals in low field, around 172 ppm, can be attributed to carboxyl groups present in uronic (glucuronic and galacturonic) and pyruvic acids. The region between 60 to 77 ppm corresponds to other carbons (C-6, C-5, C-4, C-3 and C-2) from the monosaccharides units, that, when involved in glycosidic linkages, are shifted down field (~79–84 ppm).

In summary, according to these spectroscopic chemical characterizations, the organization of these groups appears to be associated with emulsification properties of acidobacterial EPSs produced by WH15 and 5B5 strains. Additionally, these observations are in agreement with our previous results of EPS monomer characterization (Table 1).

### Rheological properties

**Figure 2:**
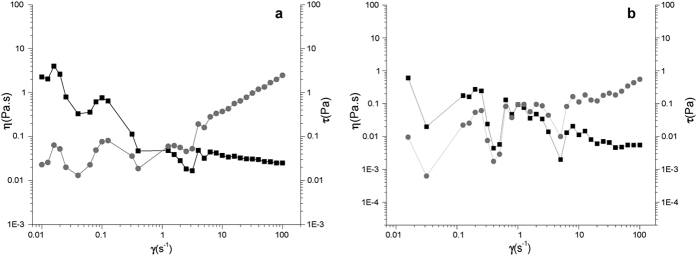
Rheological profile of acidobacterial exopolysaccharide solutions (10 g·L^−1^) (**a**) EPSWH15 and (**b**) EPS5B5. The flow curves were measured at 25 °C. The ▪ and ⦁ symbols represent η (Pa·s) and *t* (Pa) at 10 g·L^−1^, respectively.

EPS viscosity is an important parameter for industry applications. The rheological profile of EPSWH15 and EPS5B5 water solutions (10 g·L^−1^) are presented in Fig. 2. Both solutions showed non-Newtonian behaviour. The viscosities of both solutions at shear rate of 25 s^−1^ at 25 °C were low, with 31.1 and 7.1 mPa·s for EPSWH15 and EPS5B5, respectively. The viscosities of both acidobacterial EPSs decreased as shear rate increased to slightly thixotropic behaviour. Viscosity of the samples was also time dependent, increasing with commencement of mechanical stress and decreasing at the end of the process. Thus, viscosity tests were performed *versus* time at constant shear rate. At a shear rate of 10 s^−1^, the apparent viscosities of 1% (w/v) solutions of EPSWH15 and EPS5B5 were low, confirming the overall low viscosity of the samples (data not shown). The solutions may undergo physical or chemical changes such as gelation during the flow. An example of EPS as a pseudoplastic type of fluid with thixotropic behaviour was previously reported^46^ for 0.25% (w/v) *Rhizobium* EPS solution.

The values of the power-law parameters of both EPSs obtained by linear regression are shown in Table 2. As *n* tends to 1, the shear-thinning properties become less pronounced, so that Newtonian behaviour is achieved when *n* = 1. In any case, all dispersions characterized did not exhibit a marked shear-thinning response (n > 0.5). However, the acidobacterial EPSs showed lower consistency coefficients when compared to xanthan gum (2.96 ± 0.035).

### Emulsifying activity

The ability of EPS to emulsify lipids is a desired property with potential industrial and ecological applications. The commercial usage of EPS includes its application as an emulsion-forming agent (*e.g.* for bioremediation of oil polluted soil and water, food and cosmetic applications, agrochemicals), or as a stabilizer (*e.g.* for the food industry). Therefore, we compared the emulsifying ability of acidobacterial EPSWH15 and EPS5B5 with the synthetic surfactants Tween 80 and xanthan (a successfully commercialized and widely used EPS).

Emulsification indices measured for EPSWH15 and EPS5B5 emulsions in the presence of various oils and hydrocarbons are shown in Table 3. Both EPSWH15 and EPS5B5 showed bioemulsification ability by forming stable emulsions with hydrocarbon mixtures (diesel and liquid paraffin oil) and vegetable oil (sunflower seed oil) as well as with aliphatic (n-hexane) and aromatic (toluene) hydrocarbons.

Both acidobacterial EPSs (EPSWH15 and EPS5B5) showed higher E_24_ values for sunflower seeds (66.98 and 67.89%, respectively) and liquid paraffin oils (58.71 and 54.18%, respectively) (Table 3). Acidobacaterial EPSs emulsification ability for sunflower seeds oil was higher than that of xanthan. However, synthetic surfactant Tween 80 was more efficient than acidobacterial EPSs and xanthan gum, with emulsification efficiencies of 100% against sunflower seed oil and 91.35% against liquid paraffin oil.

The lowest activity was observed for emulsification of toluene, for both acidobacterial EPS (values between 10.00% and 21.30%), xanthan (38.26%) and synthetic surfactant Tween 80 (46.00%). All emulsions were stable at 30 °C during 7 and 15 days of incubation. No sedimentation, flocculation or coalescence of emulsions was observed. Moreover, EPSWH15 and EPS5B5 emulsions in the presence of sunflower and paraffin liquid oils remained stable for three months, showing no sign of droplet coalescence after standing at room temperature (30 °C) (data not shown). Emulsifying and surfactant activities together have been previously described as important functional properties of bacterial exopolymers^47^.

Interestingly, EPS5B5 gave overall better or comparable E values compared to those obtained from EPSWH15. The major differences were observed for emulsification of hexane and diesel oil. Emulsification of hexane is important for the industrial water waste treatment, while diesel emulsification may be applied in oil and oil-related product removal from the environment. In this sense, diesel oil serves as a model agent/substrate for studying hydrocarbon biodegradation. It is comprised of a variety of molecules: paraffin, olefins, naphtha and aromatic compounds. Moreover, diesel engines are dominating engines in the transportation sector, which is linked to increase of pollution emission. The mixture of diesel fuel, water, and other additives reduces emissions of particulate matters and smoke as well as NOx and CO_2_.

**Figure 3:**
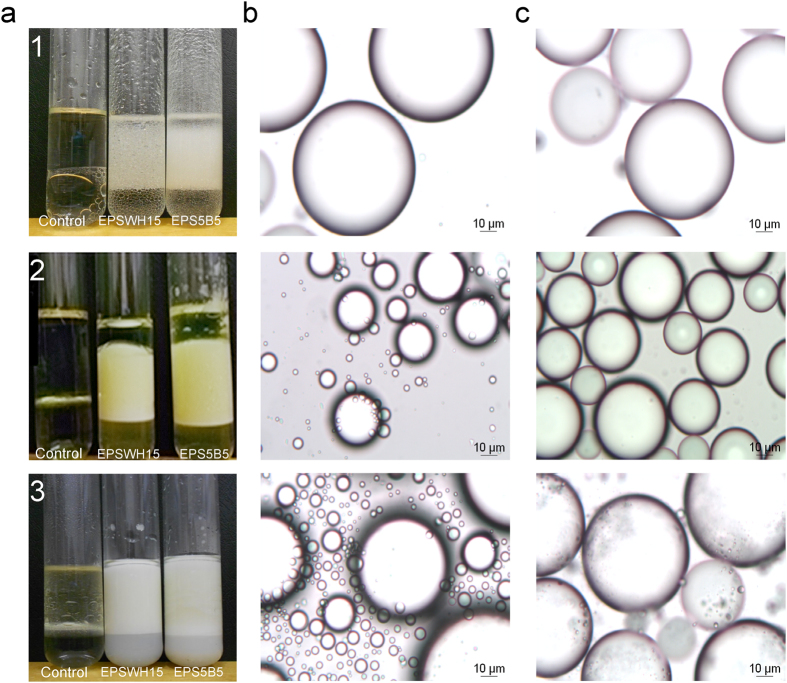
Emulsions prepared with 2 g/L of EPSWH15 and EPS5B5 with (1) liquid paraffin, (2) diesel, (3) sunflower seed oils. Panels: (a) emulsions after 24 hours of incubation at 30 °C, (**b**) droplet size distribution of emulsions prepared with EPSWH15, (**c**) droplet size distribution of emulsions prepared with EPS5B5.

An emulsion is a macroscopic dispersion of two liquids, in which one compound is a continuous part dispersed throughout small drops of the other. Among other characteristics, rheological properties, physical stability, coalescence, sedimentation, and the appearance of emulsions depend on the size and size distribution of droplets. Therefore, the size and homogeneity of emulsion droplets are important criteria for EPS industrial application. The method applied in the current study enable measurement of droplet sizes ranging from 0.02–2000 μm. The results showed that EPSWH15 and EPS5B5 produced emulsions with diesel, sunflower and liquid paraffin oils with quite small and uniform drops of oil phase (~100 μm) (Fig. 3). However, the same EPSs in the presence of hexane and toluene showed droplets of 40 μm diameter.

**Figure 4:**
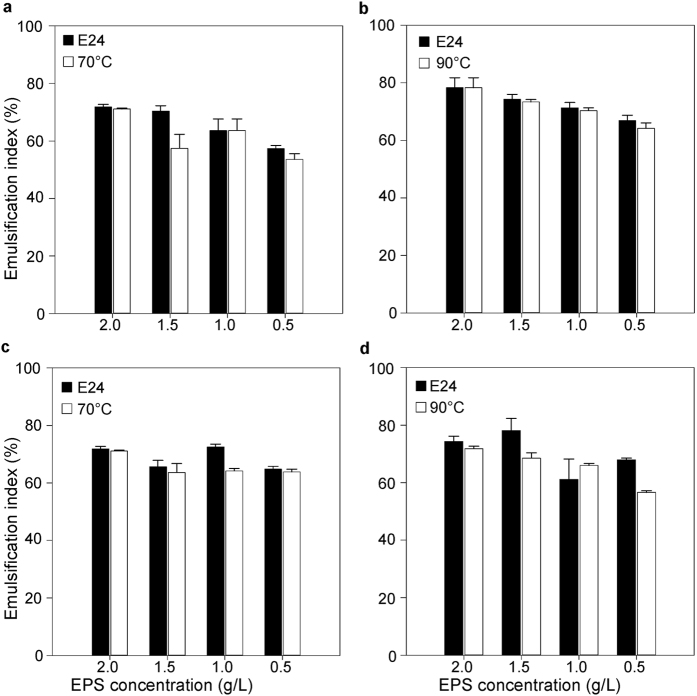
Effect of different concentrations of (**a**,**b**) EPSWH15 and (**c**,**d**) EPS5B5. Emulsification index (%) was calculated after 24 hours incubation of mixtures of different concentrations of EPS with liquid paraffin oil at 30 °C (black bars), after additional 50 min at 70 °C and at 90 °C (white bars). Errors bars represent standard error for three replicates.

The effect of acidobacterial EPS concentration on emulsion stability was studied for liquid paraffin oil. For EPSWH15, we observed a trend of increased emulsifying activity with increased polymer concentration (Fig. 4). However, those changes were not significant. EPS5B5 showed the best emulsifying activity at a concentration of 1.5 g·L^−1^. For the remaining tested concentrations, the emulsifying indexes (E) were slightly lower with no significant differences between EPS concentrations.

The stability of the EPS solutions (0; 0.5; 1.0; 1.5 and 2.0 g·L^−1^), as bioemulsifiers at temperatures of 70 °C and 90 °C for 50 min, showed that heat treatment did not reduce the emulsion forming capacity of either EPS in the presence of liquid paraffin oil (Fig. 4). Thus, the acidobacterial EPSs are thermostable as bioemulsifiers. However, most of the microbial EPSs described in the literature are not thermostable, except alasan produced by *Acinetobacter radioresistens* KA53^48^. The high stability of both EPSWH15 and EPS5B5 with regard to temperature and time exposure clearly demonstrate their potential for applications involving extreme environmental conditions.

## Conclusions

In this study, the acidobacterial EPSs of WH15 and 5B5 strains belonging to *Granulicella* sp. were characterized. The EPS of WH15 and EPS of 5B5 strains, named EPSWH15 and EPS5B5, respectively, were characterized as heteropolysaccharides containing mannose, glucose, galactose and xylose as the major monosaccharide components. Furthermore, the EPSs had better bioemulsifier properties than xanthan. The high stability of the both EPSs regarding temperature and time exposure clearly demonstrates their potential for applications in extreme environmental conditions.

The characteristics we observed for EPSs produced by *Granulicella* sp. strains WH15 and 5B5 suggest them as potential candidates for further studies of environmental, bioremediation and biotechnological applications.

## Methods

### Acidobacteria strains and culture conditions

Two *Acidodobacteria* strains, WH15^49^ and 5B5 (KM979383), were used in this study. Both *Acidobacteria* strains were characterized by sequencing the 16 S rRNA gene using the primers 27 f (5’AGAGTTTGATCMTGGCTCAG3’) and 1100r (5’AGGGTTGGGGTGGTTG3’)^50^. The two strains are genetically closely related (95% identity), belonging to genus *Granulicella,* family *Acidobacteriaceae*, order *Acidobacteriales*, class *Acidobacteriia*. For the EPS production, the strains were grown on modified PSY (pH 5.0) agar plates. The detailed composition of this cultivation medium is not available as the formula is under patent restriction (registration PI0304053-4). Inoculated plates were incubated at 30 °C for 6 days prior to EPS isolation.

### EPS extraction

EPS was collected and purified from WH15 and 5B5 *Acidobacteria* strains grown on solid medium. Cellular biomass was harvested and EPS was extracted^32^. In brief, collected bacterial biomass was suspended in ultrapure water in a ratio 2 mL of water per cellular biomass collected from one Petri dish (90 mm diameter). The resulting suspension volume was mixed with one volume of 2 M NaOH and incubated at 4 °C for 24 hours. Cells debris were removed by centrifugation at 12,000× *g* at 4 °C for 30 min. Trichloroacetic acid (TCA) was added (20% w/v) to the supernatant to remove proteins. The solution was incubated on ice for 30 min. and centrifuged (12,000× g) at 4 °C for 1 hour. The supernatant containing soluble EPS was collected, and 3 volumes of cold 96% (v/v) ethanol were added. The mixture was placed at -20 °C for 24 hours to precipitate exopolysaccharides and remove proteins and nucleic acids. The mixture was then centrifuged (12,000× g, 4 °C, 30 min.) and the EPS pellet was washed three times with 80% (v/v) ethanol. The EPS weight (grams of EPS per Petri dish of culture medium) was determined after drying it in an Eppendorf Vacufuge Plus vacuum concentrator (Eppendorf, Hamburg, Germany).

### Characterization of EPS solubility

The solubility of EPS (0.5% w/v) in water and various solvents (chloroform, methanol and toluene; 99.5%) was tested by mixing dry EPS and water or solvents in 2 ml tubes. Samples were mixed by vortexing for 60 sec. The solubility was examined after 24 hours of incubation at 30 °C.

### Characterization of EPS composition

The total neutral carbohydrate content of EPSs of both bacterial strains was measured by modified Dubois *et al*.^51^ method. Briefly, 200 μl of 2% EPS aqueous solution (w/v) was mixed with 600 μl of concentrated sulphuric acid. The samples were mixed by vortexing for 10 min followed by addition of 120 μl of 5% phenol aqueous solution (pH 5.6). The mixture was heated at 90 °C for 5 min. After cooling at room temperature for 5 min, the absorption was measured at wave length of 490 nm (DU 640 Spectrophotometer, Beckman).

The protein content was measured by Bradford assay^52^ using bovine serum albumin (BSA, Merck) as a standard, as well as determined by UV spectrophotometry (Nanodrop ND-1000, Thermo Scientific).

### Determination of EPS homogeneity and molecular weight (M_w_)

Lyophilized samples (1 mg of total sugar) of the *Acidobacteria* strains WH15 and 5B5 were dissolved in water (1 mL), filtered through a Millipore^®^ membrane (0.22-μm pore size) and injected (200 μL) into a high performance size exclusion chromatography (HPSEC) column coupled to a refractive index (RI) detector model RID 10 A, and UV-Vis detector (Shimadzu Co., Kyoto, KYT, Japan). An Ultrahydrogel column (7.8 × 300 mm) system (Waters) with exclusion limit of 7 × 10^6^, 4 × 10^5^, 8 × 10^4^ and 5 × 10^3^ Da arranged in series was used. The mobile phase was 0.1 M NaNO_3_ with sodium azide 0.03%, and a flow rate 0.6 mL/min. A standard curve of dextrans with M_w_ of 1400, 1100, 670, 500, 410, 266 and 150 kDa was made to determine the Mw for EPS samples. Data analysis was performed using LC solution software (Shimadzu Corporation).

### Modelling of composition by monosaccharide type for EPSWH15 and EPS5B5

Using our experimentally determined monomeric molecular masses (~1.4 MDa) and relative monosaccharide abundances for both EPSs, we calculated a multiplicative factor n for each EPS according to Eq. 1: 1,400,000 (Da) ≤ n[Σ(Mi)(ai)], where n is an integer multiplicative factor (the independent variable), and Mi and ai are the molecular mass and experimentally determined relative abundance, respectively, for each monosaccharide type i. For EPSWH15, n = 86 was the lowest integer value that fulfils Eq. 1. For EPS5B5, n = 387 was the lowest integer value that fulfils Eq. 1. For each ~1.4 MDa sized EPS, the approximate number of monosaccharides for each monosaccharide type i was calculated as n·ai rounded to the nearest integer value.

### Infrared (FTIR) spectroscopy

Freeze-dried EPS (1 mg) were pressed to a pellet in a 16 mm diameter mould. Fourier transform infrared (FTIR) spectra were acquired by an Attenuated Total Reflectance-Fourier Transform Infrared Spectrometer (ATR-FTIR) (Bruker-VERTEX 70, Germany), with a single reflection diamond crystal. The absorbance range was 4000–400 cm^−1^, 4 cm^−1^ of spectral resolution, and 32 scans were averaged. The spectra were baseline corrected from 3900 to 2800 cm^−1^ and from 1800 to 900 cm^−1^ and normalized to the peak intensity at 1050 cm^−1 ^53.

### Nuclear magnetic resonance (NMR) data acquisition

One-dimensional ^1^H and solid-state ^13^C NMR spectra of both EPSs were acquired using a 9.4 T Varian Inova 400 spectrometer. The solid state spectra were acquired using a Variable Amplitude Cross Polarization Magic Angle Sample Spinning sequence (VACP-MAS). The ^1^H π/2 pulse was pw = 4 μs, contact time ct = 1 ms, acquisition time at = 13.8 ms, recycle time rt = 3 s, decoupling bandwidth db = 60 kHz, spectral width sw = 40 KHz. For ^1^H NMR spectra, the sample was hydrolysed with 2 mol L^−1^ trifluoroacetic acid (TFA) by boiling sealed ampoules at 121 °C for 2 h. For ^13^C NMR spectra, the samples (200 mg) were packed in a 5 mm zirconia rotor and spun at magic angle at 9 kHz. The spectra were averaged (10,000 scans) and filtered by an exponential function (line broadening factor of 20 Hz).

### EPS monosaccharide composition characterization

One mg of each EPS was hydrolysed with 2 mol L^−1^ TFA by boiling sealed ampoules at 120 °C for 2 h; each raw EPS preparation was analysed by RP-HPLC using the 1-phenyl-3-methyl-5-pyrazolone monomer chemical identification methodology described by Fu and Oneill^54^. Prepared samples were applied for monosaccharide identification by reversed-phase chromatography (RP-HPLC) system equipped with a model SPD-M10A VP photodiode-array (PDA) detector (Shimadzu Scientific Instruments, Kyoto, Japan). The monitoring wavelength was 245 nm. 1-phenyl-3-methyl-5-pyrazolone (PMP) labeled derivatives of monosaccharides were used as standards [monosaccharides D-glucose (Glc), D-galactose (Gal), D-galacturonic acid (GalA), L-rhamnose (Rha), D-mannose (Man) and Xylose (Xyl) (Sigma Chemical GmbH, Deisenhofen, Germany), and glucuronic acid (GlcA) (Fluka Chemika GmbH, Germany)] and the identification method was according to Fu & Oneill^52^. Sample aliquots (20 μl) were injected into the HPLC system by the automatic autosampler injector (Shimadzu, model SIL-10ADvp). The analysis was performed in triplicates.

### Rheological properties of EPS in aqueous solution

The EPS was suspended in ultrapure water (1%, w/v) and incubated at 20 °C for 24 h to ensure full hydration. The EPS suspension response to applied forces was measured by controlled stress rheometer (Rheometrics Scientific). The rheological tests were conducted at 25 °C in triplicates. The ranges were determined using a shear rate control experiment in which the maximum shear rate value was 100 s^−1^.

The consistency index ‘K’ and the flow behaviour index ‘n’ were determined from the power law model^55^ given by the equation η = Kγ^(n−1)^, where η is the apparent viscosity (Pa·s), γ is the shear rate (1/s) and ‘n’ is the flow behaviour index (dimensionless). The value of ‘n’ was obtained from the slope of the log-log plot of viscosity *versus* shear rate. The value of ‘K’ was calculated from the intercept of the same graph.

### Emulsifying activity

The emulsifying activity of EPSs in various oils (sunflower seed, diesel and liquid paraffin) and hydrocarbons (toluene and hexane) was analysed by using the method of Freitas *et al*.^25^ modified by Castellane *et al*.^56^. Controls included water as negative control, and Tween 80 and Xanthan gum as positive controls. Briefly, EPS or xanthan aqueous solutions (2% w/v) were added to each of the hydrophobic compounds (3:2 ratio, v/v) and vortexed for 2 min. The test was performed in a sterile screw cap glass tube (100 mm × 13 mm). The emulsion indexes (E) of different oils and hydrocarbons were determined after 24, 168 and 360 hours by calculating the formula: E = (h_E_/h_t_) × 100, where h_E_ is the height of the emulsion layer and h_t_ is the total height of the liquid. All tests were performed in triplicate.

### Effect of EPS concentration and temperature on emulsion stability

In order to test the effect of EPS **c**oncentration on the emulsion stability, the most promising emulsion based on the E indexes was selected. The new emulsion was prepared in different concentration of EPS (0; 0.5; 1.0; 1.5 and 2.0 g·L^−1^). The emulsion indexes (E) were calculated after 24 hours of incubation at 30 °C. Additionally, to test the thermal stability, the emulsions were heated to 70 °C and 90 °C for 50 min. After cooling down to 30 °C and incubated for 2 h, the emulsification capacity was measured and compared to the corresponding values before the heat treatments.

### Estimation of emulsion droplet size distribution

The size of droplets formed during the emulsification process was evaluated after 24 h incubation at 30 °C. The size of the emulsion droplets was determined by photography using a Leica microscopy equipped with a Leica DPD 250 camera (400× overall magnification). Emulsion was placed into measuring unit with deionized water as dispersant. The experimental error of our optical procedure for drop-size determination is estimated to be around 0.3 μm, which allows the use of the microscopy data as a reference for the drop-size estimation of the studied emulsions.

### Statistical analysis

Emulsifying activity of each EPS in various oils and hydrocarbons were analysed by one-way ANOVA using R version 3.1.2. The effects of EPS concentration and heat (temperature) on emulsion stability were analysed by two-way ANOVA. The differences among means were detected by the Tukey’s post hoc test at 95% significance level. All analyses were conducted on normalized data.

## Additional Information

**How to cite this article**: Kielak, A. M. *et al*. Characterization of novel *Acidobacteria* exopolysaccharides with potential industrial and ecological applications. *Sci. Rep.* **7**, 41193; doi: 10.1038/srep41193 (2017).

**Publisher's note:** Springer Nature remains neutral with regard to jurisdictional claims in published maps and institutional affiliations.

## Supplementary Material

Supplementary Information

## Figures and Tables

**Table 1:** Monosaccharide composition of the exopolysaccharides EPSWH15 and EPS5B5 produced by *Acidobacteria* WHT15 and 5B5 strains, respectively.

Exopolysaccharide	Man	Glc	Gal	Xyl	Rha	GlcA	GalA
	mean ± SD (%)^a^						
EPSWH15 (Rel. abundance)^b^	10.25 ± 0.02 (1.000)	13.55 ± 0.08 (1.322)	26.12 ± 0.06 (2.548)	41.81 ± 0.03 (4.079)	0.065 ± 0.01 (0.006)	8.09 ± 0.09 (0.789)	0.085 ± 0.02 (0.008)
EPS5B5 (Rel. abundance)^b^	47.85 ± 0.06 (1.000)	4.01 ± 0.05 (0.084)	26.45 ± 0.09 (0.553)	14.14 ± 0.09 (0.296)	4.93 ± 0.08 (0.103)	1.50 ± 0.04 (0.031)	* (*)

^a^For n = 3 biological replicates.

^b^For each EPS, numbers in parentheses are relative abundances for each monosaccharide relative to mannose.

Man, mannose; Rha, rhamnose; GlcA, glucuronic acid; GalA, galacturonic acid; Glc, glucose; Gal, galactose; Xyl, xylose; *, undetected at a sensitivity limit of 0.001%.

**Table 2:** Coefficients of the power law model for acidobacterial EPSWH15 and EPS5B5 solutions (10 g·L^−1^).

Type of exopolysaccharide	K	η
Xanthan	2.96 ± 0.035	0.22 ± 0.07
EPSWHT15	0.06 ± 0.002	0.82 ± 0.08
EPS5B5	0.03 ± 0.001	0.57 ± 0.04

Mean values ( ± standard deviation); *n* = Flow behavior index; K = consistency coefficient obtained by the Ostwald-de Waele model: η = Kγ^(n−1)^.

**Table 3:** Emulsification activity of 2% (w/v) EPS solutions from *Acidobacteria* strains WH15 (EPSWH15) and 5B5 (EPS5B5), Tween 80 and xanthan with different oils and different hydrocarbons in 3:2 ratio (v/v).

Oil/Hydrocarbon	Positive Controls^a^				Acidobacterial Exopolysaccharides^a^					
	Tween 80	Xanthan			EPSWH15			EPS5B5		
	E_24_	E_24_	E_168_	E_360_	E_24_	E_168_	E_360_	E_24_	E_168_	E_360_
Sunflower seed oil	100 ± 0.02a*	**50.03 ± 2.43a**	44.70 ± 2.40	30.56 ± 2.78	**66.98 ± 1.45a**	63.00 ± 2.41	62.04 ± 1.60	**67.89 ± 4.18a**	64.57 ± 1.85	66.65 ± 0.93
Diesel oil	**63.21 ± 2.96b**	**76.85 ± 4.24b**	67.84 ± 8.24	66.02 ± 6.67	12.28 ± 1.75b	12.28 ± 1.75	14.43 ± 2.98	33.65 ± 2.83b	33.65 ± 2.83	32.45 ± 4.08
Liquid paraffin oil	**91.35 ± 3.42a**	**70.81 ± 12.36ab**	39.00 ± 3.23	37.12 ± 4.46	**58.71 ± 0.65c**	49.50 ± 2.95	54.13 ± 1.39	**54.18 ± 3.66a**	52.78 ± 2.78	51.68 ± 9.52
Toluene	46 ± 1.54bc	38.26 ± 7.88ac	29.10 ± 1.85	9.08 ± 1.50	10.19 ± 1.60b	10.29 ± 1.70	11.17 ± 2.56	21.30 ± 1.60b	21.48 ± 1.28	21.30 ± 1.60
Hexane	49 ± 2.45bc	22.51 ± 12.42c	23.15 ± 1.60	7.41 ± 1.60	26.85 ± 1.60d	26.85 ± 1.60	25.93 ± 1.16	**59.26 ± 8.93a**	58.33 ± 4.81	50.92 ± 4.24

^a^Results are expressed as percentages of the total height occupied by the emulsion; Values in bold: Emulsifier Index (E24) with values above 50%; Mean values of three replicates ( ± standard deviation). *Treatments with the same letter do not differ by the by the Tukey’s post hoc test (*P* < 0.05).

E_24_ emulsification index after 24 h; E_168_ emulsification index after 168 h; E_360_ emulsification index after 360 h.
