# Supplementary material for: Characterization of novel Acidobacteria exopolysaccharides with potential industrial and ecological applications
Source: Sci Rep. 2017 Jan 24;7:41193. doi: 10.1038/srep41193 (PMC5259719; doi:10.1038/srep41193)
Supplement: Supplementary Information [file srep41193-s1.pdf]

Characterization of novel *Acidobacteria* exopolysaccharides with potential industrial and ecological applications

Anna M. Kielak[1*], Tereza C. L. Castellane[2*], Joao C. Campanharo[2], Luiz A. Colnago[3], Ohana Y. A. Costa[1], Maria L. C. Silva[4], Johannes A. van Veen[1], Eliana G. M. Lemos[2] & Eiko E. Kuramae[1#]

[1]Netherlands Institute of Ecology (NIOO-KNAW), Department of Microbial Ecology, P.O. Box 50, 6700 AB, Wageningen, the Netherlands. [2]São Paulo State University (Unesp), School of Agricultural and Veterinarian Sciences, Rod. Prof. Paulo Donato Castellane km 5, CEP 14884-900, Jaboticabal, Brazil. [3]Embrapa Instrumentação, Rua XV de Novembro, 1452, CEP 13560-970, São Carlos, Brazil,. [4]Faculdade de Ciências e Tecnologia (Unesp), Departmento de Química e Bioquímica, Rua Roberto Símonsen, 305, CEP 19060-900, Presidente Prudente, Brazil.

*Equal contribution

#Corresponding author: E. E. Kuramae

e-mail: e.kuramae@nioo.knaw.nl

Tel/FAX: +31 (0)317 473 493

Netherlands Institute of Ecology (NIOO-KNAW)

Droevendaalsesteeg 10

6708 PB Wageningen, The Netherlands

Running title: Novel Acidobacterial exopolysaccharide

Supplementary Material

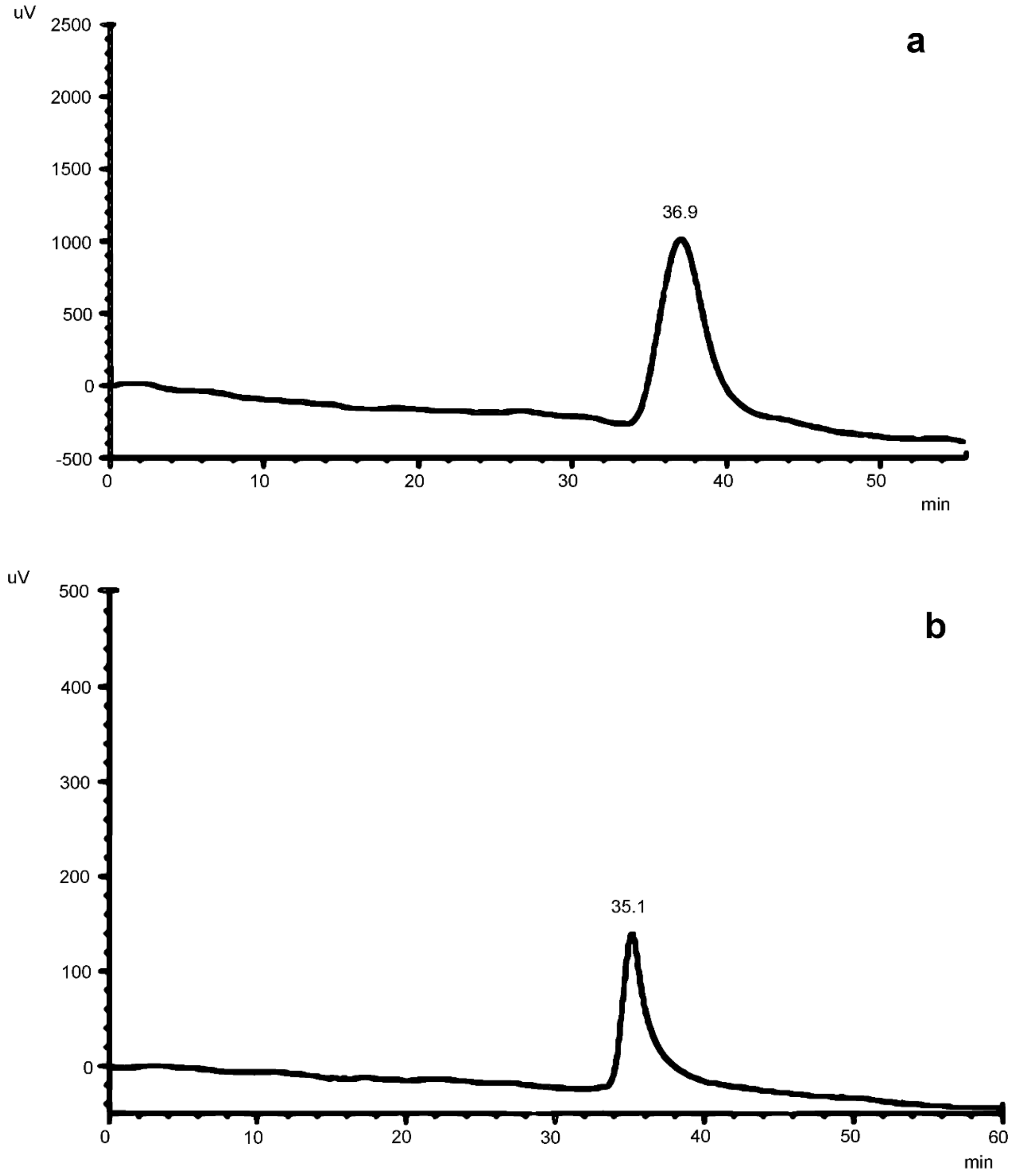

Fig. S1. High performance size-exclusion chromatography (HPSEC) equipped with a refractive index detector (RID) analysis of the *Acidobacteria* strains WH15 (a) and 5B5 (b).

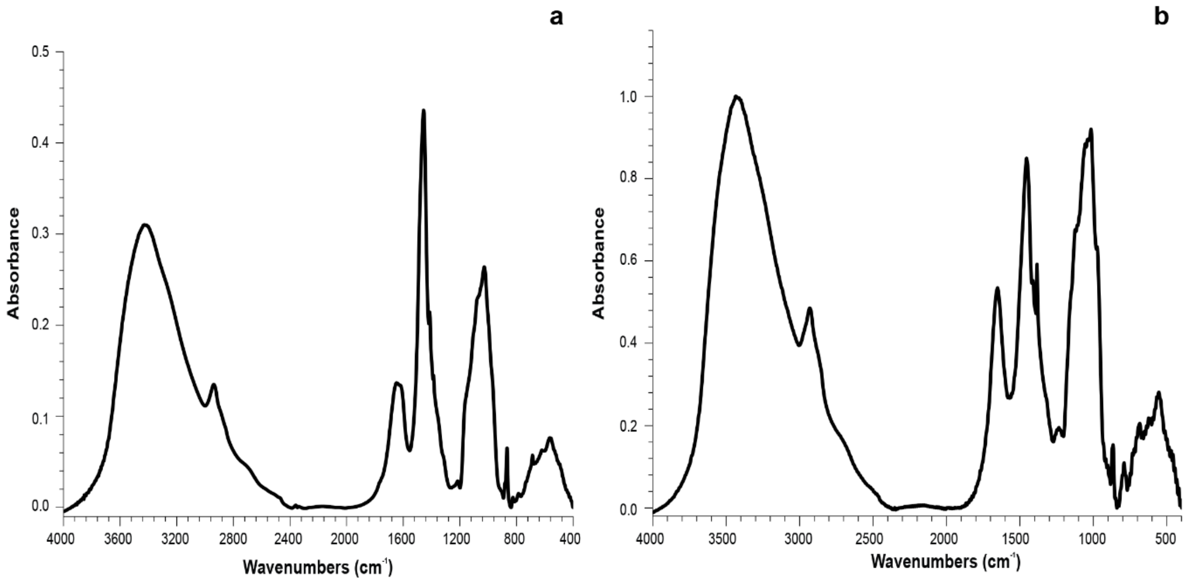

Fig. S2. Fourier transform infrared (FT-IR) spectra of exopolysaccharide produced by *Granulicella* sp. (a) WH15 and (b) 5B5 strains. These are original absorbance spectra. All spectra normalized by the absorption band at 1050 cm^{-1}.

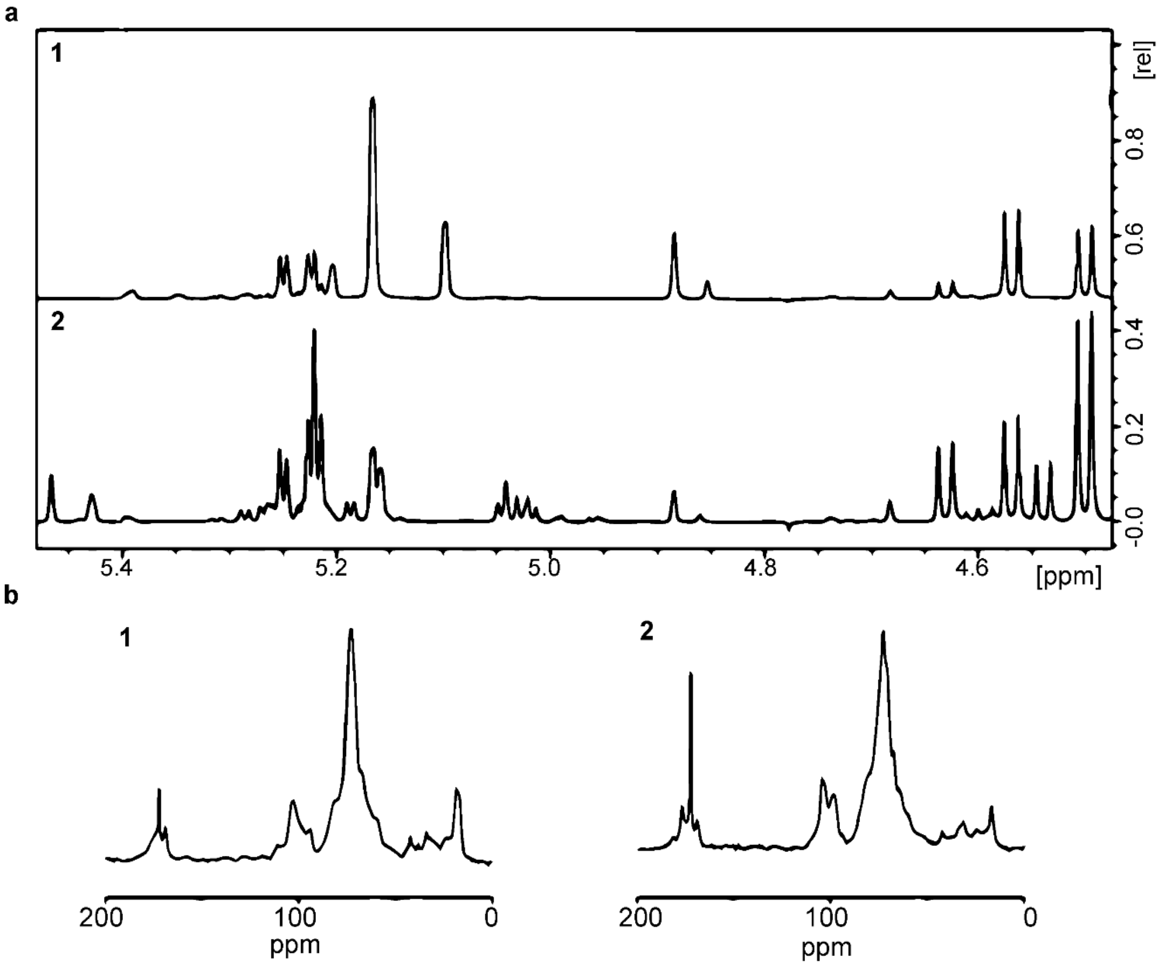

Fig. S3. Spectra of the EPS produced by *Granulicella* sp. (1) 5B5 and (2) WH15 strains. (a) ^{1}H NMR spectrum (b) ^{13}C NMR spectrum.
